# Gastric transit and small intestinal transit time and motility assessed by a magnet tracking system

**DOI:** 10.1186/1471-230X-11-145

**Published:** 2011-12-29

**Authors:** Jonas Wors&#216e, Lotte Fynne, Tine Gregersen, Vincent Schlageter, Lisbet A Christensen, Jens F Dahlerup, Nico JM Rijkhoff, Søren Laurberg, Klaus Krogh

**Affiliations:** 1 Department of Surgery P, Aarhus University Hospital, Aarhus, Denmark; 2 Center for Sensory-Motor Interaction (SMI), Department of Health Science and Technology, Aalborg University, Aalborg, Denmark; 3 Neurogastroenterology Unit, Department of Hepatology and Gastroenterology V, Aarhus University Hospital, Aarhus, Denmark; 4 Motilis Medica SA, Lausanne, Switzerland; 5 Department of Hepatology and Gastroenterology V, Aarhus University Hospital, Aarhus, Denmark

## Abstract

**Background:**

Tracking an ingested magnet by the Magnet Tracking System MTS-1 (Motilis, Lausanne, Switzerland) is an easy and minimally-invasive method to assess gastrointestinal transit. The aim was to test the validity of MTS-1 for assessment of gastric transit time and small intestinal transit time, and to illustrate transit patterns detected by the system.

**Methods:**

A small magnet was ingested and tracked by an external matrix of 16 magnetic field sensors (4 × 4) giving a position defined by 5 coordinates (position: **x, y, z, and angle: θ, ϕ)**. Eight healthy subjects were each investigated three times: (1) with a small magnet mounted on a capsule endoscope (PillCam); (2) with the magnet alone and the small intestine in the fasting state; and (3) with the magnet alone and the small intestine in the postprandial state.

**Results:**

Experiment (1) showed good agreement and no systematic differences between MTS-1 and capsule endoscopy when assessing gastric transit (median difference 1 min; range: 0-6 min) and small intestinal transit time (median difference 0.5 min; range: 0-52 min). Comparing experiments (1) and (2) there were no systematic differences in gastric transit or small intestinal transit when using the magnet-PillCam unit and the much smaller magnetic pill. In experiments (2) and (3), short bursts of very fast movements lasting less than 5% of the time accounted for more than half the distance covered during the first two hours in the small intestine, irrespective of whether the small intestine was in the fasting or postprandial state. The mean contraction frequency in the small intestine was significantly lower in the fasting state than in the postprandial state (9.90 min^-1 ^vs. 10.53 min^-1^) (p = 0.03).

**Conclusion:**

MTS-1 is reliable for determination of gastric transit and small intestinal transit time. It is possible to distinguish between the mean contraction frequency of small intestine in the fasting state and in the postprandial state.

## Background

The prevalence of gastrointestinal motility and functional gastrointestinal disorders is high in the general population [1,2]. Furthermore symptoms of disturbed GI motility are often a significant problem in patients with other medical problems. Diagnosing and alleviating these disorders require good evaluation methods that can identify abnormal GI physiology. Gastrointestinal motility is usually described in terms of regional transit times or as intraluminal pressure changes. Scintigraphy is the gold standard for determination of gastric emptying and small intestinal transit [3,4]. Contraction patterns have been investigated using manometry catheters. Solid state catheters with small pressure transducers have facilitated ambulatory examinations and allowed recording of diurnal variation [5-7]. Disadvantages of these techniques include the invasiveness, the exposure to radiation and that they are relatively expensive. The hydrogen breath test is an alternative for determination of transit times, but it is affected by small intestinal bacterial overgrowth and does not distinguish between gastric and intestinal transit times [8].

New techniques aim to improve the quality of motility data and also to reduce the side effects and patient discomfort. Video capsule endoscopy, primarily used for evaluation of the small intestinal mucosa pathology, may be an alternative for determination of transit times [9,10]. However, for the mere purpose of obtaining transit times, it is expensive and analysis is time consuming. Computerized picture analysis of capsule endoscopy images has recently been used for description of small intestinal motility patterns [11]. Lately, a wireless motility capsule (Smartpill) that measures temperature, pressure and pH has been used to investigate segmental and whole-gut transits [12,13]. Magnetic resonance imaging have also been used to measure gastric and small intestinal motility [14,15]. MRI has also been used to track the position of fluorine labeled capsules giving information about small intestinal motility patterns and this can be combined with anatomical data [16].

Information about motility patterns and transit can also be obtained by tracking a small magnet through the gastrointestinal tract. Early methods based on ingestion of a small solid magnet have been refined to improve spatial and temporal resolutions [17-21]. High resolution data on gastrointestinal transit have been obtained using multi-channel superconducting quantum interference, but the equipment is expensive and requires a shielded environment [22-24]. Magnetic moment imaging using a tracking system with anisotropic magneto-resistor sensors was recently validated with scintigraphy demonstrating good correlation between gastric transit time and positional data [25]. The Magnet Tracking System (MTS-1; Motilis, Lausanne, Switzerland) has been developed for use in a standard laboratory setting [26,27]. MTS-1 has been used in animal studies, studies in healthy humans, and in patients with neurogenic bowel dysfunction [28-33]. However, a validation with simultaneous measurements using established methods is needed. If the difference in contraction frequency and position of the magnet measured with MTS-1 can be used to determine pyloric and ileocecal passages, then MTS-1 will be an easy, minimally-invasive, and non-radiant tool to provide valid information on gastric transit time and small intestinal transit times.

The primary aim of this study was to investigate if MTS-1 could be used to reliably determine gastric transit and small intestinal transit time. Data from simultaneous capsule endoscopy was used as reference. Furthermore, small intestinal motility patterns recorded with MTS-1 in the fasting state and in the postprandial state were compared for identification of migrating motor complex phase III during fast. An algorithm was applied for classification of fast movements, slow movements, and very slow movements and by comparing small intestinal contraction frequencies.

## Methods

### Subjects

Eight healthy volunteers (3 males, median age 30 years; range: 25-61 years), with median BMI 21.3 kg m^-2 ^(range: 20.2-26.5 kg m^-2^) were included. No subjects had undergone abdominal surgery, were taking medication or suffered from diseases affecting gastrointestinal motility. All participants signed informed written consent and the study was approved by the local scientific ethical committee (M-20080037).

### Magnet Tracking System, MTS-1

**Figure 1:**
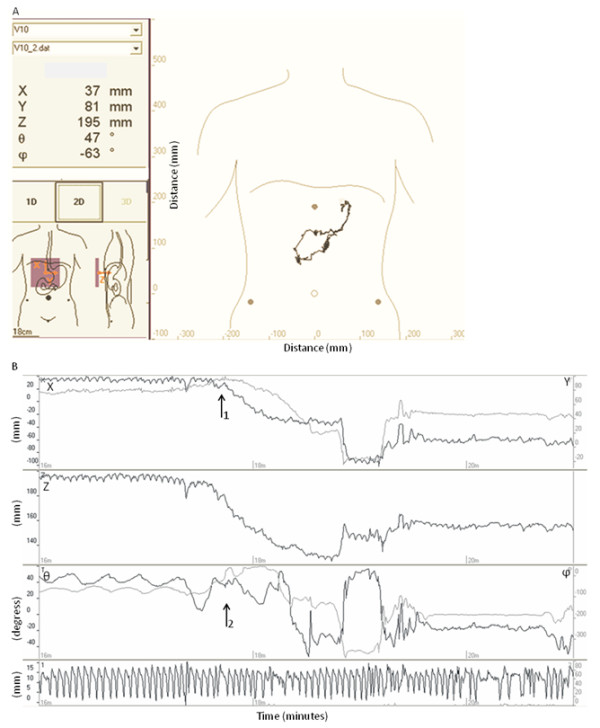
**Real time recording with the MTS-1 (example from one subject)**. 1A: The position x, y and z and orientation θ and ϕ are displayed. Position of the sensor array over the body is seen to the left. To the right a recording of the movement through the duodenal arch is displayed. 1B: Duodenal passage (from 17 m 40 s to 19 m 30 s) is seen as a change in position (x, y and z) (arrow 1) and disappearance of the characteristic 3 contractions min^-1 ^pattern of the stomach (θ and ϕ) (arrow 2). The curve at the bottom shows noise from respiration and movement.

Subjects ingested a small magnetic pill (dimensions: 6 × 15 mm, weight: 0.9 g, density: 1.8 g cm^-3^, magnetic moment 0.2 Am^2^), which was tracked by a matrix of 4 × 4 magnetic field sensors separated by 5 cm and placed over the abdomen. The position of the sensor matrix with respect to anatomical landmarks was noted (iliac spines, intercostal angle, pubic bone) (Figure 1). With a sampling rate of 10 Hz, each sensor measured the magnetic induction dependent on the distance between the sensors and the magnetic pill and the orientation of the pill. The position and orientation of the magnetic pill was defined by 5 coordinates (position: x, y, z, and angle: θ, ϕ). The change in position coordinates reflected propagation of the magnet. The change of the angles reflected change in orientation, which correlated with the contraction frequency of the relevant gastrointestinal segment. Data were analysed on a computer running custom-made software (MTS_Record, Motilis, Lausanne, Switzerland) showing real-time position and orientation of the magnetic pill (Figure 1). Before the measurements began, the sensors were calibrated by offsetting the earth's magnetic field. Artefacts due to respiration and movement were recorded using accelerometers placed on the abdomen and the neck. During post processing, an adaptive algorithm was used to filter out movements in phase with the respiration.

### MTS-1 combined with PillCam

**Figure 2:**
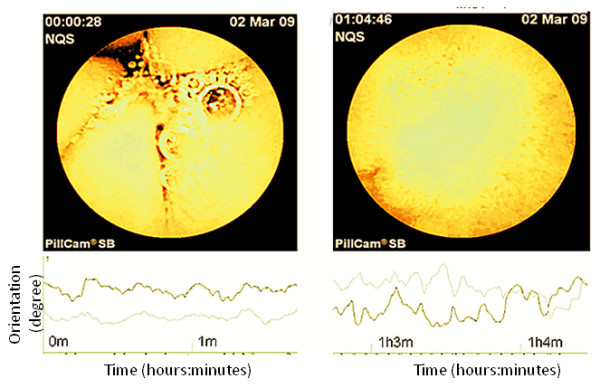
**Correlation of anatomical data and motility data using the PillCam and the Magnet Tracking System, Left: image from the stomach with simultaneous MTS-1 data (orientation θ and ϕ on the y-axis) showing a contraction frequency of approximately 3 min^-1 ^(arbitrary unit), consistent with localization in the stomach**. Right: image from the proximal small intestine with simultaneous MTS-1 data showing a contraction frequency of approximately 9-10 min^-1 ^(arbitrary unit) consistent with localization in the small intestine.

The validity of gastric transit and small intestinal transit determined with MTS-1 was tested through comparison with the simultaneous use of a PillCam (Figure 2). The video capsule (PillCam, Given, Yoqnaem, Israel) measures 11 × 26 mm and contains an imaging device (field of view of 156°) and a light source at one end of the capsule [34]. Images were transmitted at a rate of two images s^-1 ^with a battery powered light source lasting for a minimum of eight hours.

A magnet-PillCam unit was constructed by gluing (Loctite 4013 medical line, Henkel, Rocky Hill, CT, USA) the magnetic pill on a PillCam and covering the magnet-PillCam unit with a polyurethane sheet.

### Protocol

The subjects underwent three experiments on three separate days, all starting at 9 AM and continuing for six to eight hours with the following steps: (1) ingestion of the magnet-PillCam unit where a standard meal (≈1500 kJ, 32% fat) was given after four hours investigation in the fasting state with the investigation continued until ileocecal passage; (2) ingestion of the magnetic pill alone in a similar setting as (1); and (3) ingestion of the magnetic pill followed by a standard meal given right after pyloric passage (≈ 2200 kJ, protein, 48% fat). During the investigations, subjects were placed in a bed with head elevation (> 45°) or lying down. They were encouraged not to talk or move. The recordings were interrupted for small breaks on request.

### Data analysis

Experiment (1) was used to test the validity of MTS-1 for assessment of gastric transit and small intestinal transit time. Experiments (1) and (2) were used to compare gastric transit and small intestinal transit of two different sized objects. Experiments (2) and (3) were used to compare the fasting and the postprandial motility patterns for two hours after pyloric passage.

**Figure 3:**
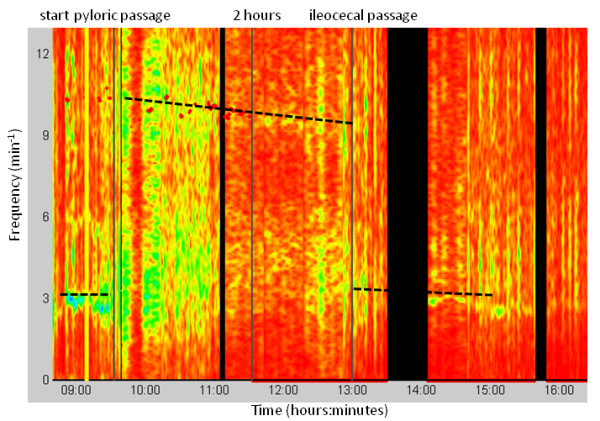
**Time frequency plot**. The contraction frequencies (dotted line) are illustrated as a function of time. A dominant frequency of 3 min^-1 ^is seen initially as the magnet pill is located in the stomach. At approximately 09:45 the magnetic pill enters the small intestine and the dominant frequency changes to 10 min^-1^. Ileocecal passage is seen at approximately 13:00 as a drop in frequency to 4-5 min^-1^. The green color indicates contractions with high amplitudes at a given frequency and the red color indicates contractions with lower amplitudes. The red dots are peak amplitudes obtained when the magnet is performing very slow movements.

**Figure 4:**
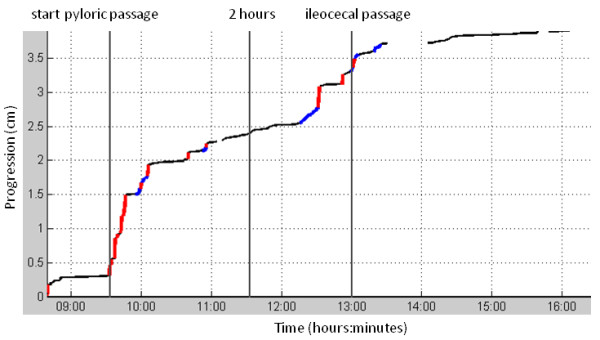
**Progression of the magnetic pill over time during fast**. Investigation made in fasting conditions. Pyloric passage, 2 hour after pyloric passage, and ileocecal passage is marked. The color of the plot represents different velocities (red: > 15 cm min^-1^, blue: < 15 cm min^-1^, black: < 1.5 cm min^-1^). The contractions frequency can only be calculated when progression is very slow (< 1.5 cm min^-1^). Most distance through the small intestine is covered during the period just after pyloric passage and during the period just before ileocecal passage. These two periods, separated by approximately 90 minutes, probably reflect phase III of the MMC.

Two investigators independently determined the gastric transit and the small intestinal transit time in each investigation, and the mean times were used for further comparisons. The gastric transit time was defined as the time from ingestion of the magnetic pill until pyloric passage. The cessation of the 3 contractions min^-1 ^pattern, typical for the stomach, the appearance of the duodenal arch, and the beginning of the 8-11 contractions min^-1 ^of the small intestine were the hallmarks of pyloric passage (Figure 1). Small intestinal transit was determined as time from the pyloric passage until the ileocecal passage, which was identified as cessation of the 8-10 min^-1 ^contraction frequency of the small intestine(Figure 3), the occurrence of a short fast movement (Figure 4), and the magnetic pill situated in the lower right quadrant. The contraction frequencies were displayed in a time-frequency plot with a color code indicating the contraction amplitude. These data were obtained by computing the short-time Fourier transform (STFT) (Figure 3). For this purpose, custom made software was used (MTS_Tool, Motilis, Lausanne, Switzerland). A standard approach for analysis of time-frequency maps was used. The power spectral density is estimated by and fast fourier transform on a short segment of data. A time frame of 3 min was used, and a Hamming window was applied. Calculations for the sliding window were conducted every 10 samples giving a time-frequency map. At each instant, peaks detection is applied to select main present frequencies. Only steady values were considered and extreme values were omitted based on Bayesian algorithms.

Capsule endoscopy with PillCam was used as the gold standard for detection of pyloric and ileocecal passage. Using PillCam images, gastric transit time was defined as time from ingestion of the magnet-PillCam unit until the time of the first picture in the duodenum. Small bowel transit was defined as the time from pyloric passage until the first picture of ileocecal passage. The PillCam recordings were examined by two experts and the mean value of their results was used as reference.

**Figure 5:**
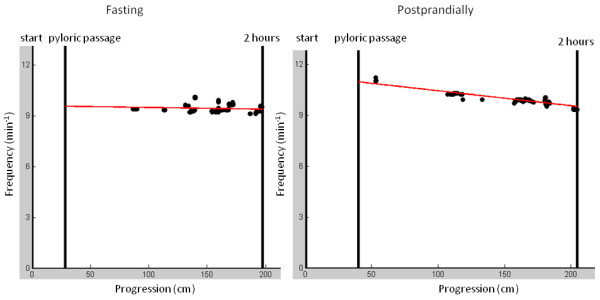
**Small intestinal contraction frequency during fast and postprandially (example from investigations in one subject)**. The progression in the small intestine is on the x-axis, and the contraction frequency in the small intestine for two hours after pyloric passage is on the y-axis. In general, the mean contraction frequency was lower during fast compared to postprandially (9.48 min^-1 ^vs. 10.25 min^-1^). The decrease in contraction frequency per 2 hours was smaller during fast when compared to postprandially (-0.18 Hz cm^-1 ^vs. -1.45 Hz cm^-1^).

Motility patterns were analysed with Motilis-dedicated software for the upper gastro-intestinal tract (MTS_Tool, Motilis, Lausanne, Switzerland). The mean small intestinal propagation velocity for two hours following pyloric passage was computed. The mean contraction frequencies of the stomach and the small intestine were calculated using the contractions with the highest amplitudes obtained when the magnet was performing very slow movements (i.e. when there was no progression of the magnet). The mean contraction frequency in the small intestine was calculated using only contractions with a frequency higher than 6 min^-1^. The frequency peaks were selected using a convolution of the fast fourier transform with the "shape of a peak" described by a Gaussian function. The frequencies obtained during progression of the magnet were discarded, while frequencies obtained when the magnet did not progress were included. With this approach the Doppler effect (contraction frequency as a function of velocity of the magnet) was evaded. A linear regression was used to derive the change in instantaneous contraction frequency during the first two hours after pyloric passage (Figure 5).

An initial analysis of velocity histograms identified a trimodal distribution of velocities and the cut offs were made to separate the three types of movements velocities: fast movements (> 15 cm min^-1^), slow movements (between 1.5 and 15 cm min^-1^), and very slow movement (< 1.5 cm min^-1^). Based on this analysis, an algorithm was developed for automatic classification of movements in the small intestine [29].

### Statistics

Numerical data are given as means and standard deviations and non-gaussian distributed data are given as medians and total range. Statistical significance was tested with Wilcoxon's test (non-parametric test for paired data), and the level of significance was set at 0.05.

## Results

### Inter observer variation

For the MTS-1 investigations, the median difference between the two observers' determination of transit times was 1 min (range: 0-11 min) for gastric transit and 6 min (range: 0-33 min) for small intestinal transit.

### Validation of gastric transit and small intestinal transit data determined with MTS-1

In all subjects, the magnet-PillCam unit was easily ingested and passed the cardia within 30 s. No pathology was seen in the stomach or in the small intestine. Pyloric passage was identified with the PillCam in all eight subjects. In one subject the magnet-PillCam unit underwent numerous regurgitations forth and back in the pyloric region before definitive pyloric passage. Agreement between gastric transit times determined with MTS-1 (median 56 min; range: 5-133 min) and with PillCam (median 57.5 min; range: 7-127 min) was good with a median difference of 1 min (range: 1-6 min) with no systematic difference (Table 1).

**Table 1:** Gastric transit and small intestinal transit times obtained using the magnet-PillCam unit and the magnetic pill in eight subjects

Subject ID	Magnet-PillCam unit				Magnetic pill alone	
	
	PillCam		MTS-1		MTS-1	
	
	Gastric transit (min)	Small intestinal transit (min)	Gastric transit (min)	Small intestinal transit (min)	Gastric transit (min)	Small intestinal transit (min)
1	127	-	133	-	73	402

2	29	241	30	241	53	251

3	19	292	20	284-294	4	260

4	60	307	60	255	52	-

5	7	275	5	276	48	292

6	55	209	53	209	17	261

7	107	245	107	245	23	-

8	60	398	59	398	18	241

Median	57.5	275	56	255	35.5	260.5

The magnet-PillCam unit was ingested during fast and a meal was given after four hours. In subject number four, ileocecal passage took place during a ten minutes break. Ileocecal passage determined with capsule endoscopy took place after eight minutes into the break, and error of 8 min was used for comparison with the PillCam. In three of sixteen investigations, ileocecal passage did not occur during the eight hour protocol.

Ileocecal passage was identified using PillCam in seven subjects. In one subject, ileocecal passage could not be identified during the eight-hour investigation. Usually the magnet-PillCam unit was situated in the terminal ileum for a length of time (median 57 min; range: 19-148 min) before ileocecal passage. The small intestinal transit time determined with MTS-1 (median 255 min; range: 209-398 min) and PillCam (median 275 min; range: 209-398 min) showed good agreement as the median difference was 1 min (range: 0-52 min) with no systematic difference (Table 1).

### Fasting and postprandial propagation velocities in the small intestine

The two-hour motility data during fasting and postprandially are presented in table 2. The median two-hour propagation velocity was 2.2 cm min^-1 ^(range: 1.1-2.8 min) during the fasting, and 2.3 cm min^-1 ^(range: 1.7-3.8 min) postprandially (p = 0.50). Most small intestinal transit occurred through very fast movements (> 15 cm min^-1^) accounting for median 60% (range: 34-62%) of the distance in median 3% (range: 2-4%) of the time during the fast. Likewise in the postprandial state, 60% (range: 42-74%) of the distance occurred with very fast movements in median 3% (range: 2-7%) of the time.

**Table 2:** Fasting and postprandial motility for two hours after pyloric passage

Subject ID	Fasting								Postprandial							
	
	Fast movements (> 15 cm min^-1^)		Slow movements (< 15 cm min^-1^)		Very slow movements (< 1.5 cm min^-1^)		Mean contraction frequency (min^-1^)	Mean progression velocity (cm min^-1^)	Fast movements (> 15 cm min^-1^)		Slow movements (< 15 cm min^-1^)		Very slow movements (< 1.5 cm min^-1^)		Mean contraction frequency (min^-1^)	Mean progression velocity (cm min^-1^)
	(cm)	(min)	(cm)	(min)	(cm)	(min)			(cm)	(min)	(cm)	(min)	(cm)	(min)		
1	111	4	33	17	43	99	9.78	1.6	91	3	79	36	18	81	10.32	1.6

2	100	4	29	12	40	104	9.48	1.4	71	2	79	36	18	82	10.25	1.4

3	58	2	14	10	22	108	9.32	0.8	97	3	21	8	46	109	10.72	1.4

4	162	5	77	35	42	80	10.27	2.3	143	5	11	6	41	109	9.33	1.6

5	56	2	43	19	14	99	10.14	0.9	83	4	59	27	30	89	10.56	1.4

6	79	3	108	44	45	73	9.92	1.9	219	8	85	42	39	70	11.04	2.9

7	65	2	45	20	34	98	10.14	1.2	108	5	31	16	39	99	11.00	1.5

8	95	4	49	20	11	96	10.15	1.3	88	3	6	4	25	113	11.02	1.0

Median	87	3.5	44	19.5	37	98.5	9.90	1.4	94	3.5	45	21.5	34.5	94	10.53	1.5

Progression (cm) and duration (min) of fast (> 15 cm min-1), slow (between 1.5 and 15 cm min-1), and very slow (< 1.5 cm min-1) movements during fast and after a standard meal. The mean progression velocities during two hours are also given.

### Transit and small intestinal motility patterns of magnetic pill versus magnet-PillCam unit

For the magnetic pill, median gastric transit time was 35.5 min (range: 4-73 min); median small intestinal transit time was 260.5 min (range: 241-402 min) (table 1). This finding did not differ significantly from transit times of the magnet-PillCam unit (p = 0.21, p = 0.89). There was no significant difference between the median two-hour propagation velocity with the magnetic pill (median 1.3 cm min^-1^; range: 0.8-2.3 min) and the larger magnet-PillCam unit (median 1.5 cm min^-1^; range: 1.0-1.7 min) (p = 0.89). In one subject, there was a difference of 52 min between small intestinal transit determined with capsule endoscopy and MTS-1. In subject number four, ileocecal passage occured during a 10 min break. Ileocecal passage determined with capsule endoscopy occurred after an 8 min of the break, so a maximum error of 8 min was used for calculation. In two of the investigations with the magnetic pill and in one of the investigations with the magnet-PillCam unit, the ileocecal passage did not occur during the eight-hour investigation (Table 1).

### Frequency of contractions

The mean contraction frequency of the stomach was 2.85 ± 0.29 min^-1^. Movements through the duodenum were fast (mean propagation velocity: 28 cm s^-1 ^± 20 cm s^-1^) and often separated by one or two pauses. The mean contraction frequencies in the small intestine was 9.90 ± 0.14 min^-1 ^for two hours during fast and 10.53 ± 0.16 min^-1 ^postprandially (p = 0.03). The mean contraction frequency decreased during the first two hours after pyloric passage both during fasting and postprandially. Compared with postprandially (-1.12 min^-1 ^cm^-1^), the slope during fasting was less step (-0.49 min^-1 ^cm^-1^) (p = 0.04) (Figure 5).

## Discussion

The MTS-1 is a non-radiant and minimally invasive tool to determine gastrointestinal transit times. MTS-1 is accurate for determination of colorectal transit time, and pilot data on gastric and small intestine contraction patterns and transit times have been published [29,30]. In the present study we found that MTS-1 is valid for determination of gastric transit and small intestinal transit times. The inter-observer variation for assessment of gastric transit was low and not expected to be clinically relevant. Video capsule endoscopy was used as the "gold standard", and agreement between the two methods was good. Estimates of pyloric and ileocecal passage were based on the position of the magnet pill in the frontal plane and changes in the frequency spectrum as a function of time. The latter were recognizable and characteristic for the stomach, the small intestine, and the colon. An algorithm for analysing the time frequency plots may allow development of automatic determination of gastric transit and small intestinal transit time.

The precision of MTS-1 depends on the position and orientation of the magnet with respect to the sensor matrix. With only one sensor positioned 100 mm from the magnetic pill, the positioning error in the frontal plane is 10 mm, however changes in orientation of only 1-2 degrees can be detected [27]. This error is reduced by adding more sensors in a matrix, and the currently used system can track the magnetic pill at distances of more than 200 mm. The absolute accuracy of MTS-1 is approximately 1-2 cm, which is sufficient for anatomical localization. The amplitude of small back and forth movements can be measured more accurately (1-2 mm, rotation of 0.5°). With good quality recordings of the respiratory rhythm, the correction of respiratory artifacts at all amplitudes was accurate. A problem with the MTS-1 is that movement of the small intestine inside the abdomen affects the measurements. This can only be overcome with simultaneous collection of anatomical data (computer tomography), not included in this protocol. Thus, the distance covered and the velocity of the magnet pill reflects movement of the intestine as well as antegrade and retrograde intraluminal movement. The former is probably of minor importance but given the lack of distinction between back and forth mixing movements and short regular antegrade movements, velocity of the magnet pill should be considered a motility index rather than an estimate of progression through the intestine. However, most of the distance was covered during fast or very fast movement and those were clearly identifiable. A shortcoming of our protocol was that short breaks were allowed during the investigation, potentially influencing measurements of distance and calculation of total distance travelled in the small intestine. However, using the positioning of the sensor with respect to anatomical landmarks indicated that this error was very little.

Compared to scintigraphy, MTS-1 has no risk of radiation exposure; this is especially important if children are investigated. Scintigraphy, however, allows determination of gastric emptying for both solids and liquids (i.e. meals and macronutrients), whereas magnetic tracking only determines transit of the magnetic pill, since a small solid will leave the stomach with a phase III MMC [35]. Given the size of the magnet pill it is possible that its passage through the small intestine will differ from the passage of a meal. The same holds true for other methods including the wireless motility capsule (Smartpill) and the PillCam. Future comparison with scintigraphy may clarify this aspect. In the present study the meal was given to induce the postprandial small intestinal motility pattern when the magnet pill had reached the doudenum. The postprandial state affects the whole small intestine and we, therefore, consider the observed differences between fast and postprandial states valid even if the magnet pill did not behave entirely like the meal.

Capsule endoscopy has been used to assess small intestinal motility [11]. However, the size of the PillCam may affect contractions and transit [36]. Data from the present study seem to contradict this, as transit times with the specially constructed magnet-PillCam unit did not differ from those obtained with the much smaller magnetic pill.

Antroduodenal and small intestinal manometry is used clinically in the evaluation of patients with suspected severe dysmotility such as chronic intestinal pseudoobstruction [5,37]. It was anticipated that MTS-1 could be used for identification of phase III in the migrating motor complexes (MMC) and in the recordings during fasting we saw several examples of suggested MMC phase III (Figure 4). However, no statistical difference in the distribution of fast movements which could represent phase III MMC was seen when comparing fasting and postprandial motility data. Future studies combining manometry and MTS are needed to validate changes in MMC seen by MTS. The propagated distance of the magnetic pill was the same during fasting and postprandially. During fasting the contraction frequency decreased in the aboral direction; this finding was even more pronounced postprandially, which likely reflects the small intestine adapting to intake of food and to promoting absorption. Similarly, the mean contraction frequency in the small intestine increased postprandially. A linear fitting was used to analyse contractions in the small intestine. It is recognised, that this model does not take into account the magnets progression velocity changes along the small intestine. Also, only data obtained when the magnet was performing very slow movements were included explaining why more data points exist at the end of the two-hour period. With further improvement of the analyses, it may become possible to identify motility patterns with pathological significance.

Recently, the wireless motility capsule (Smartpill, SmartPill Corporation, Buffalo, NY, USA) has been introduced. It is for ambulatory use, and measures pressure, pH, and temperature throughout the gastrointestinal tract [35,38]. The Smartpill provides reliable information about gastric transit, small intestinal transit, total colonic transit, and some contraction patterns [12,13]. It is correct that most parameters obtained with MTS are also available with the SmartPill. Also, the SmartPill is developed into a clinically useful design which the MTS is not. There are two major differences: 1). SmartPill detects pressure whereas MTS detects movement. Measuring pressure in a moving object where one does not know the direction of the pressure sensor according to the lumen, direction of movement, or bowel wall involves major sources of error. These are avoided by MTS. 2). SmartPill determines total colorectal transit time, but allows no estimation of right versus left colonic transit. MTS tracks position and thereby potentially allows determination of segmental colonic transit. This may be clinically important.

In general, variations in gastric transit time are large and account for much of the variation in oro-cecal transit time [39]. Accordingly, we found large inter-subjective variations in gastric transit and small intestinal transit times. This finding is mainly caused by lack of timing of magnetic pill ingestion with phase III of MMC.

## Conclusion

1MTS-1 system is a promising minimally-invasive, non-radiant research tool for the investigation of gastrointestinal motility. The method is accurate for the assessment of gastric transit and small intestinal transit times. Furthermore, it was possible to distinguish between fasting and postprandial small intestinal mean contraction frequencies, which warrants further exploration.

## Competing interests

JW, LF, TG, LAC, JFD, NJ, SL, and KK all declare that they have no competing interests.

VS is a shareholder in Motilis.

## Authors' contributions

JW: study design, conduction of experiments, data analysis, preparation of draft, and approval of the final manuscript, LF: study design, conduction of experiments, and approval of the final manuscript, TG: study design, conduction of experiments, data analysis, and approval of the final manuscript, VS: data analysis and approval of the final manuscript, LAC: data analysis (capsule endoscopy) and approval of the final manuscript, JFD: data analysis (capsule endoscopy) and approval of the final manuscript, SL: study design and approval of the final manuscript.

KK: study design, data analysis, preparation of draft, and approval of the final manuscript, NJMR: data analysis, preparation of draft, and approval of the final manuscript.

## Pre-publication history

The pre-publication history for this paper can be accessed here:

http://www.biomedcentral.com/1471-230X/11/145/prepub
